# Early-life determinants of hypoxia-inducible factor 3A gene (*HIF3A*) methylation: a birth cohort study

**DOI:** 10.1186/s13148-019-0687-0

**Published:** 2019-07-01

**Authors:** Toby Mansell, Anne-Louise Ponsonby, Vania Januar, Boris Novakovic, Fiona Collier, David Burgner, Peter Vuillermin, Joanne Ryan, Richard Saffery, Barwon Infant Study Investigator Team, Peter Vuillermin, Anne-Louise Ponsonby, John Carlin, Katie Allen, Mimi Tang, Richard Saffery, Sarath Ranganathan, David Burgner, Terry Dwyer, Kim Jachno, Peter Sly

**Affiliations:** 1 Murdoch Children’s Research Institute, Parkville, Australia; 2 Department of Paediatrics, University of Melbourne, Parkville, Australia; 3 The Florey Institute of Neuroscience and Mental Health, Parkville, Australia; 4 School of Medicine, Deakin University, Geelong, Australia; 5 Child Health Research Unit, Barwon Health, Geelong, Australia; 6 Department of Paediatrics, Monash University, Clayton, Australia; 7 School of Public Health & Preventive Medicine, Monash University, Melbourne, Australia

**Keywords:** *HIF3A*, DNA methylation, SNPs, Pregnancy, Infant, Gestational diabetes, Pre-eclampsia

## Abstract

**Background:**

Methylation of the hypoxia-inducible factor 3α gene (*HIF3A*) has been linked to pregnancy exposures, infant adiposity and later BMI. Genetic variation influences *HIF3A* methylation levels and may modify these relationships. However, data in very early life are limited, particularly in association with adverse pregnancy outcomes. We investigated the relationship between maternal and gestational factors, infant anthropometry, genetic variation and *HIF3A* DNA methylation in the Barwon Infant Study, a population-based birth cohort. Methylation of two previously studied regions of *HIF3A* were tested in the cord blood mononuclear cells of 938 infants.

**Results:**

No compelling evidence was found of an association between birth weight, adiposity or maternal gestational diabetes with methylation at the most widely studied *HIF3A* region. Male sex (− 4.3%, *p* < 0.001) and pre-eclampsia (− 5.4%, *p* = 0.02) negatively associated with methylation at a second region of *HIF3A*; while positive associations were identified for gestational diabetes (4.8%, *p* = 0.01) and gestational age (1.2% increase per week, *p* < 0.001). *HIF3A* genetic variation also associated strongly with methylation at this region (*p* < 0.001).

**Conclusions:**

Pre- and perinatal factors impact *HIF3A* methylation, including pre-eclampsia. This provides evidence that specific pregnancy complications, previously linked to adverse outcomes for both mother and child, impact the infant epigenome in a molecular pathway critical to several vascular and metabolic conditions. Further work is required to understand the mechanisms and clinical relevance, particularly the differing effects of in utero exposure to gestational diabetes or pre-eclampsia.

**Electronic supplementary material:**

The online version of this article (10.1186/s13148-019-0687-0) contains supplementary material, which is available to authorized users.

## Background

Evidence suggests that risk factors for a range of metabolic and cardiovascular diseases begin very early in life, including in utero [1]. In turn, the in utero environment is sensitive to maternal environmental exposures [2], potentially mediating these effects. This is described by the Developmental Origins of Health and Disease (DOHaD) concept, which postulates that the early-life environment is important in shaping later adult health and risk of disease [3].

The biological mechanism(s) underlying the influence of prenatal exposures on neonatal health and adult disease are poorly understood but are thought to be mediated, at least in part, by epigenetic processes, including DNA methylation [2]. In adults, both genome-wide and gene-specific methylation have been associated with adiposity-related measures, including body mass index (BMI), waist circumference and levels of inflammatory markers [4–11]. A cross-sectional epigenome-wide association study (EWAS) in adult blood identified DNA methylation of three CpG sites in the first intron of some transcript variants of the hypoxia-inducible factor 3α (HIF-3α) gene (*HIF3A*) in association with BMI [6], subsequently replicated by two independent cross-sectional studies using adult blood [12, 13].

The family of hypoxia-inducible factors, including HIF-3α, are believed to play key roles in angiogenesis, metabolism and obesity [14–16] and variation in DNA methylation of the associated CpG sites (identified in adipose tissue and whole blood) has been linked with altered gene expression [6] and adiposity measures in several subsequent studies [4, 7–11, 17–21]. The majority of previous findings are cross-sectional and have investigated methylation in blood, adipose tissue, or umbilical cord (Additional file 1). However, one longitudinal study in children found evidence for early BMI predicting later *HIF3A* methylation in blood [19]. Emerging evidence also suggests an influence of gestational diabetes [22] and maternal pre-pregnancy BMI on cord blood methylation at a second *HIF3A* promoter region [19]. Despite these findings, the tissue specificity and direction of causality at the two regions of *HIF3A* in newborns remain generally unclear.

Here, we aimed to investigate (1) the relationship between maternal factors in pregnancy and *HIF3A* methylation at two gene regions, (2) the relationship between infant anthropometry and *HIF3A* methylation, (3) the influence of *HIF3A* genetic variation on methylation, and (4) the dependence of each of these influences on *HIF3A* methylation levels.

## Results

### Cohort characteristics and methylation data

**Fig. 1:**
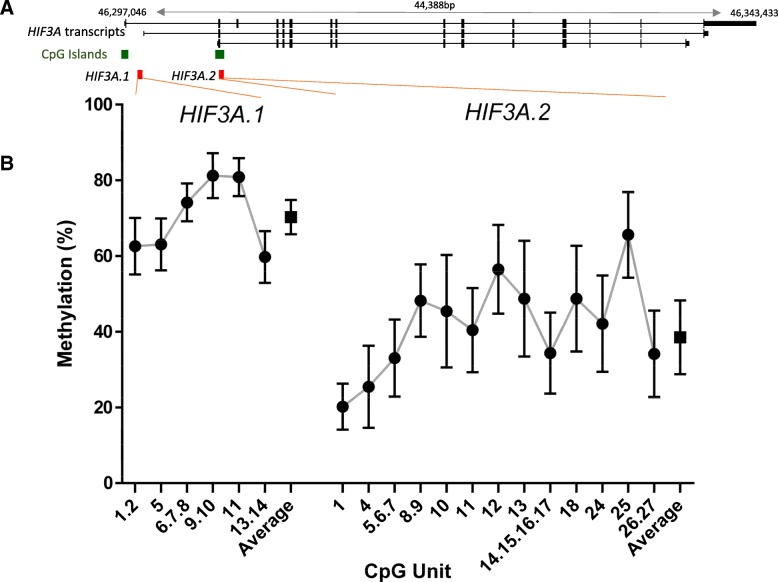
Summary of DNA methylation across the *HIF3A* regions investigated in this study. **a** Diagram of the two analysed regions (red) relative to different *HIF3A* transcripts (black) and CpG islands (green). Gene transcription is from left to right. Solid segments in the transcripts indicate exons, and the connecting lines indicate introns. **b** The distribution of DNA methylation for each CpG unit (circles) and the average methylation for each region (squares). Error bars are mean ± standard deviation

The mean age of mothers in this study at conception was 31.4 years (standard deviation (SD) 4.7) and mean pre-pregnancy BMI 25.3 (SD 5.3). The incidence of gestational diabetes (GDM) and pre-eclampsia was 5.0% (40/800) and 2.9% (27/934), respectively. Mean infant gestational age was 39.5 weeks (SD 1.4), mean birth weight 3559.6 g (SD 496.3), and 51.4% (482/938) of infants were male. Sample characteristics are shown in Table 1. The distribution of methylation for each CpG unit and the averages for the two *HIF3A* regions investigated in this study (herein referred to as *HIF3A*.*1* and *HIF3A*.*2*) are shown in Fig. 1. The mean average methylation level across *HIF3A*.*1* was 70.3% (SD 4.5), with mean methylation of individual CpG units ranging from 59.5 to 80.8%. *HIF3A*.*2* was generally less methylated, with a mean average methylation across *HIF3A*.*2* of 38.5% (SD 9.7) and mean methylation of individual CpG units between 20.2 and 66.4%. Data were approximately normal in distribution and within each region was strongly correlated (*p* < 0.0001 for all pairwise correlations, Additional file 2). As such, subsequent analyses focussed on the average methylation across each region.

**Table 1:** Cohort characteristics for the *HIF3A*.*1* and *HIF3A*.*2* samples

Characteristics	*HIF3A*.*1* sample *n* = 490	*HIF3A*.*2* sample *n* = 938
Maternal		
	Mean (SD)	Mean (SD)
Age at conception (years)	31.7 (4.4)	31.4 (4.7)
Pre-pregnancy BMI (kg/m^2^)	25. (5.1)	25.3 (5.3)
	*n* (%)	*n* (%)
Smoked during pregnancy (any)	59 (12.0)	146 (15.6)
Gestational diabetes	17 (3.9)	40 (5.0)
Pre-eclampsia	17 (3.5)	27 (2.9)
Infant		
	Mean (SD)	Mean (SD)
Gestational age (weeks)	39.5 (1.4)	39.5 (1.4)
Birth weight (g)	3548.5 (500.0)	3559.6 (496.3)
Birth weight z-score	0.4 (0.9)	0.4 (0.9)
Triceps+subscapular sum (mm)	9.7 (2.1)	9.9 (2.2)
	*n* (%)	*n* (%)
Sex (male)	241 (49.2)	482 (51.4)

*SD* standard deviation

### Pre-eclampsia and gestational diabetes associated with average *HIF3A*.*2* methylation

**Fig. 2:**
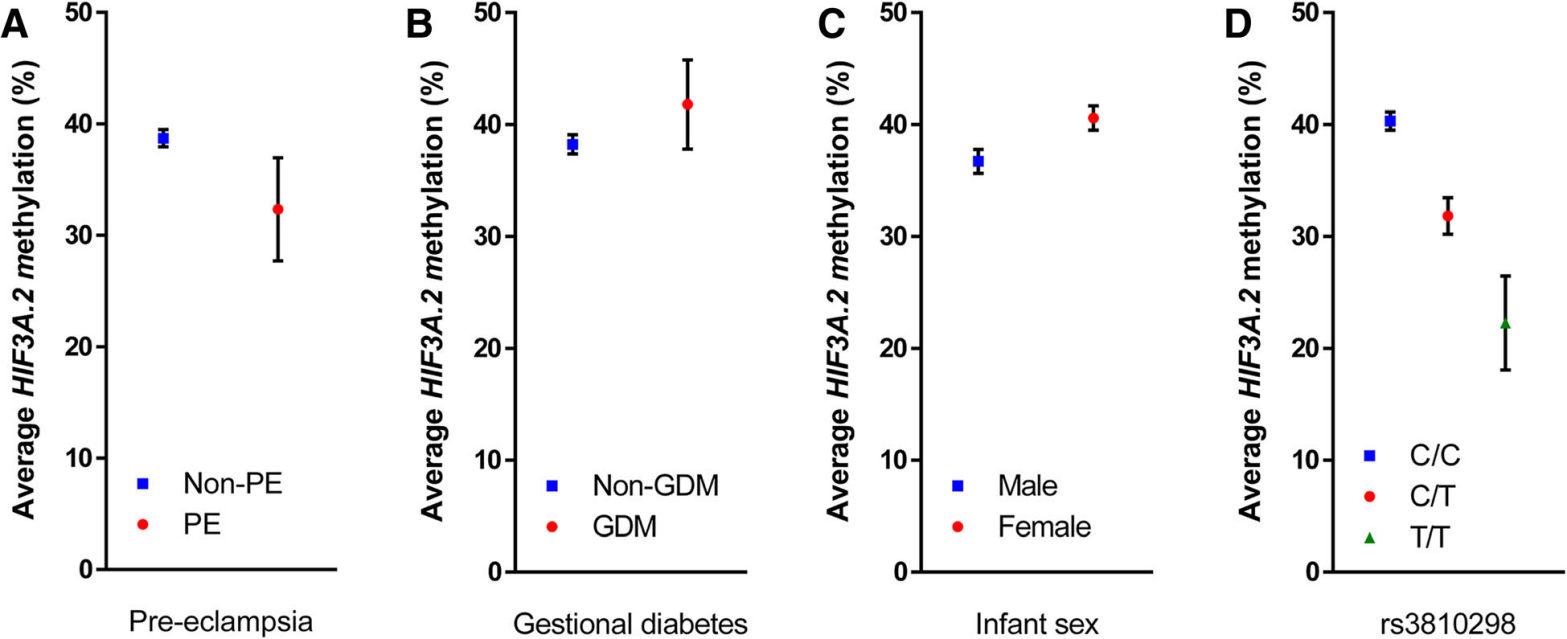
Distribution of average *HIF3A*.*2* methylation stratified by **a** pre-eclampsia (PE), **b** gestational diabetes (GDM), **c** infant sex and **d** rs3810298 genotype. Error bars are mean ± 95% confidence interval

In univariate analysis investigating the relationship between maternal factors and cord blood methylation, pre-eclampsia was associated with lower average methylation across *HIF3A*.*2* (− 6.38% methylation, 95% confidence interval (CI) − 10.94, − 1.82, *p* = 0.006), while GDM was associated with higher average *HIF3A*.*2* methylation (3.53% increased methylation, 95% CI − 0.43, 7.49, *p* = 0.08) (Table 2, Fig. 2a, b). No maternal measures were associated with average *HIF3A*.*1* methylation.

**Table 2:** Associations of cohort characteristics with average *HIF3A*.*1* and *HIF3A*.*2* methylation

Characteristics	Average *HIF3A*.*1* methylation *n* = 423		Average *HIF3A*.*2* methylation *n* = 609	
Maternal				
	*r*	*p*	*r*	*p*
Age at conception (years)	0.01	0.80	-0.01	0.76
Pre-pregnancy BMI (kg/m^2^)	-0.01	0.81	0.04	0.40
	Effect (SE)	*p*	Effect (SE)	*p*
Smoked during pregnancy (any)	0.01 (0.66)	0.99	0.63 (1.10)	0.56
Gestational diabetes	0.04 (1.19)	0.97	3.53 (2.01)	0.08
Pre-eclampsia	0.09 (1.24)	0.94	− 6.38 (2.32)	0.006
Infant				
	*r*	*p*	*r*	*p*
Gestational age (weeks)	− 0.08	0.10	0.19	< 0.001
Birth weight (g)	− 0.05	0.32	0.09	0.03
Birth weight z-score	− 0.03	0.61	0.02	0.61
Triceps+subscapular sum (mm)	− 0.01	0.89	0.03	0.54
	Effect (SE)	*p*	Effect (SE)	*p*
Sex (male)	0.78 (0.44)	0.08	− 3.87 (0.78)	< 0.001

Effect size given as difference in percentage methylation. *r* correlation coefficient, *SE* standard error. *p* values for binary measures (smoking during pregnancy, gestational diabetes, pre-eclampsia, and male infant sex) are from unpaired two-tailed Student’s *t* tests comparing the exposed group to the unexposed group. *p* values for continuous measures are from correlation coefficient tests

### Infant sex and gestational age associate with average *HIF3A*.*2* methylation; sex has weaker association with *HIF3A*.*1* methylation

Male sex of the infant was negatively associated with average *HIF3A*.*2* methylation (− 3.87% methylation compared to females, 95% CI − 5.40, − 2.34, *p* < 0.001) (Fig. 2c). Gestational age (*r* = 0.19, *p* < 0.001) and absolute birth weight (*r* = 0.09, *p* = 0.03) were positively associated with average *HIF3A*.*2* methylation (Table 2). Infant sex had a weaker association with average methylation across *HIF3A*.*1* (0.78% increased methylation in male infants, 95% CI − 0.09, 1.65, *p* = 0.08).

### Genetic influences on *HIF3A* methylation stronger at *HIF3A*.*2*

Of the 14 *HIF3A* tag SNPs considered in this analysis, rs3810298, rs112087991 and rs3826795 were strongly associated with average *HIF3A*.*2* methylation, with genotype of rs3810298 showing the greatest effect (C/T genotype − 8.48% compared to C/C, 95% CI − 10.34, − 6.63, *p* < 0.001; T/T genotype − 18.06% compared to C/C, 95% CI − 23.65, − 12.47, *p* < 0.001) (Table 3, Fig. 2d). All three of these SNPs were in strong linkage disequilibrium (Additional file 3). The genotype of rs8102595 was associated with average *HIF3A*.*1* methylation (1.40% increased methylation for heterozygote A/G genotype compared to major allele homozygote A/A, 95% CI 0.26, 2.54, *p* = 0.02 in linear regression model).

**Table 3:** Average methylation of *HIF3A*.*1* and *HIF3A*.*2* by genotype for each of the 14 tag SNPs considered in this analysis

SNP	*HIF3A*.*1* methylation (*n* = 410)			*HIF3A*.*2* methylation (*n* = 595)		
rs62111812	Mean (SD)	*n*	*p*	Mean (SD)	*n*	*p*
G/G	70.02 (4.59)	313	0.22	37.95 (9.96)	438	0.1
G/A	70.93 (4.74)	89		39.93 (8.74)	144	
A/A	71.19 (1.71)	8		39.47 (10.37)	13	
rs112087991						
T/T	70.39 (4.47)	359	0.22	39.46 (9.44)	522	< 0.0001
T/C	69.18 (5.38)	49		31.54 (8.67)	70	
C/C	69.57 (0.61)	2		27.17 (8.30)	3	
rs8102595						
A/A	69.97 (4.60)	331	0.04	38.63 (9.83)	477	0.7
A/G	71.37 (4.46)	76		37.79 (9.28)	115	
G/G	72.07 (2.20)	3		37.56 (9.56)	3	
rs3826795						
C/C	70.65 (4.18)	250	0.07	40.42 (9.14)	364	< 0.0001
C/T	69.68 (5.23)	142		35.76 (9.45)	196	
T/T	69.03 (4.16)	18		33.28 (11.52)	35	
rs3810298						
C/C	70.42 (4.27)	337	0.26	40.32 (9.02)	476	< 0.0001
C/T	69.44 (5.95)	67		31.84 (8.63)	109	
T/T	69.44 (4.36)	6		22.26 (5.88)	10	
rs140454328						
T/T	70.26 (4.61)	398	0.71	38.49 (9.72)	563	0.87
T/C	69.75 (4.01)	12		38.08 (9.90)	31	
C/C	–	0		33.83 (0)	1	
rs36063219						
C/C	70.23 (4.63)	393	0.78	38.41 (9.76)	576	0.46
C/T	70.55 (3.80)	17		40.08 (8.39)	19	
T/T	–	0		–	0	
rs3752207						
C/C	70.23 (4.43)	357	0.21	38.31 (9.54)	516	0.29
C/A	70.66 (4.95)	48		39.06 (11.02)	72	
A/A	66.83 (10.11)	5		43.75 (7.81)	7	
rs4803929						
C/C	70.13 (4.64)	355	0.04	38.6 (9.78)	510	0.29
C/T	70.7 (4.01)	53		37.41 (9.25)	83	
T/T	77.88 (4.77)	2		46.49 (8.69)	2	
rs9304657						
T/T	70.34 (4.00)	181	0.75	38.31 (10.18)	288	0.92
T/C	70.27 (5.00)	178		38.58 (9.48)	237	
C/C	69.80 (5.12)	51		38.72 (8.58)	70	
rs76789866						
T/T	70.26 (4.58)	385	0.82	38.83 (9.61)	546	0.01
T/C	70.04 (4.79)	25		34.29 (9.85)	47	
C/C	–	0		37.43 (17.94)	2	
rs75952656						
T/T	70.28 (4.67)	382	0.54	38.32 (9.70)	558	0.15
T/A	69.73 (3.29)	28		40.71 (9.81)	37	
A/A	–	0		–	0	
rs917946						
A/A	70.25 (4.58)	328	0.96	38.07 (9.51)	477	0.05
A/G	70.23 (4.72)	79		39.74 (10.47)	111	
G/G	69.44 (2.17)	3		45.28 (7.40)	7	
rs12459580						
G/G	70.08 (4.19)	119	0.65	38.71 (8.36)	168	0.93
G/C	70.44 (4.74)	212		38.36 (9.89)	295	
C/C	69.95 (4.77)	79		38.39 (10.91)	132	

*p* value is from one-way ANOVA. *SD* standard deviation

### Multivariable regression modelling for average *HIF3A*.*2* methylation

**Fig. 3:**
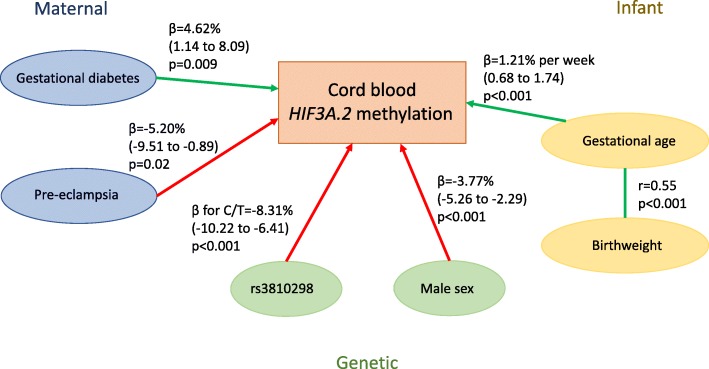
Diagram of associations between maternal, infant and genetic factors and *HIF3A*.*2* methylation in cord blood. Green arrows denote positive associations and red arrows denote negative associations. Beta values (and 95% confidence interval in brackets) and *p* values are from the final linear regression model for *HIF3A*.*2* methylation (Table 4)

Given the multiple strong associations identified by univariate analyses, more detailed regression modelling was carried out for *HIF3A*.*2*. Key covariates of GDM, pre-eclampsia, gestational age, birth weight, infant sex and rs3810298 genotype all remained independently associated with average *HIF3A*.*2* methylation (Table 4), with the exception of birth weight which attenuated after adjustment for gestational age and consequently was excluded from the final model. GDM (4.62% increased methylation, 95% CI 1.14, 8.09, *p* = 0.009) and gestational age (1.21% increased methylation per week, 95% CI 0.68, 1.74, p < 0.001) were positively associated with methylation, whereas pre-eclampsia (− 5.20%, 95% CI − 9.51, − 0.89, *p* = 0.02) and male sex (− 3.77% compared to females, 95% CI − 5.26, − 2.29, *p* < 0.001) were negatively associated with methylation. A diagram of the associations is shown in Fig. 3. Genotype at rs3810298 was not associated with any of the maternal or infant measures included in the final *HIF3A*.*2* linear regression model (Additional file 4).

**Table 4:** Final linear regression model adjusting for all key variables with average *HIF3A*.*2* methylation as outcome

	Average across *HIF3A*.*2* (*n* = 513)		
	*β* (SE)	*p*	95% CI
GDM	4.62 (1.77)	0.009	1.14 to 8.09
Pre-eclampsia	− 5.20 (2.19)	0.02	− 9.51 to − 0.89
Sex (male)	− 3.77 (0.76)	< 0.001	− 5.26 to − 2.29
Gestational age (weeks)	1.21 (0.27)	< 0.001	0.68 to 1.74
rs3810298			
C/T	− 8.31 (0.97)	< 0.001	− 10.22 to − 6.41
T/T	− 16.91 (2.73)	< 0.001	− 22.27 to − 11.54

Effect sizes (*β*) given as percentage methylation. *β* for rs3810298 categories is the difference from homozygote major allele (C/C). *GDM* gestational diabetes, *SE* standard error, *CI* confidence interval. No other SNPs were strongly associated with *HIF3A*.*2* methylation after adjustment for rs3810298, and a linear regression model with just rs3810298 accounted for a similar amount of variation in *HIF3A*.*2* methylation measures and the model with six SNPs (*R*^2^ for minimal model = 0.162, *R*^2^ for full model = 0.177, *p* = 0.29)

### Additional sensitivity analyses

Of the cellular proportions considered, granulocytes and, inversely, lymphocytes were associated with average *HIF3A*.*2* methylation (0.11% increased methylation per percentage increase in granulocytes, 95% CI 0.02, 0.20, *p* = 0.01; − 0.14% decrease per percentage increase in lymphocytes, 95% CI − 0.23, − 0.05, *p* = 0.002). Due to collinearity between granulocyte and lymphocyte proportions, the final model was adjusted for only lymphocyte proportion, which modestly decreased the association between gestational age and average *HIF3A*.*2* methylation (0.99% increased methylation per week, 95% CI 0.41, 1.57, *p* = 0.001), with no effect on other associations tested. We also examined both GDM and pre-eclampsia as a composite exposure. Methylation associated with both outcomes as mutually exclusive factors, with the GDM-only group having 4.87% higher average methylation than the non-GDM, non-pre-eclampsia group, and the pre-eclampsia-only group having 5.98% lower average methylation.

There was no evidence for interaction effects between infant sex or genotype with other key measures in terms of their effects on average *HIF3A*.*2* methylation. In the unit-specific analysis, pre-pregnancy BMI was associated with CpG11 methylation (*r* = 0.08, *p* = 0.04) (Additional file 5), but adjusting for maternal pre-pregnancy BMI did not substantially alter the estimate of effects. Similarly, the inclusion of interaction effects or pre-pregnancy BMI did not substantially improve the fit of the linear regression model. The final model applied to site-specific *HIF3A*.*2* methylation is shown in Additional file 6. We focussed on the average methylation only as no specific CpG site(s) showed evidence of a differential degree of association.

In CpG unit-specific analysis for *HIF3A*.*1*, gestational age was positively associated with methylation at CpG5 specifically (*r* = 0.12, *p* = 0.007), while maternal age at conception was weakly, positively associated with CpG5 methylation (*r* = 0.08, *p* = 0.07) (Additional file 7). Adjusting for these measures did not alter the association between infant sex and average *HIF3A*.*1* methylation. Of the cell types considered in the sensitivity analysis, activated T_reg_ cells showed the strongest evidence of an association with average *HIF3A*.*1* methylation (− 1.24% decreased methylation per activated T_reg_ percentage, 95% CI − 2.50 to 0.03, *p* = 0.06), but adjusting for cellular proportions did not alter any of the observed associations between *HIF3A*.*1* methylation and maternal and infant factors.

## Discussion

We found evidence for independent association of *HIF3A* methylation at birth with important pregnancy-related outcomes, offspring anthropometric measures and genetic variation, particularly around *HIF3A*.*2*, previously linked to maternal BMI [19]. This is consistent with multiple biological pathways intersecting through *HIF3A* epigenetic variation. In this study, our observed cord blood methylation levels were comparable with a previous cord blood study using genome-wide CpG probes across *HIF3A*.*1* and *HIF3A*.*2* [22], with *HIF3A*.*1* hypermethylated compared to *HIF3A*.*2*, which had intermediate levels of methylation. Future studies should investigate the nature of this intermediate methylation to determine whether it may be due in part to monoallelic methylation or cellular heterogeneity. Distinct environmental effects on methylation at the two regions is consistent with a previous longitudinal childhood cohort study [19] that reported a positive association between maternal pre-pregnancy BMI and cord blood methylation at CpG probes in and near *HIF3A*.*2* but not *HIF3A*.*1*, and reported a weak negative correlation in methylation between the two regions, as we have found here.

### HIF-3α regulation and function

The *HIF3A* gene produces up to eight different transcripts from potentially three different promoter regions [23], with the likelihood that *HIF3A*.*2* methylation influences expression of a subset. Hypoxia has been reported to upregulate transcripts associated with all 3 promoter regions [23]. It has previously been found the DNA methylation of *HIF3A*.*1* CpG5 is negatively associated with expression in adipose tissue [6], though a later study reported that BMI, but not methylation, is negatively associated with expression in adipose tissue [9]. The decreased methylation of *HIF3A*.*2* in response to pre-eclampsia might be anticipated to increase gene expression, which is consistent with upregulation of *HIF3A* transcription in response to hypoxia. A genome-wide study investigating associations between gene expression measured by RNA sequencing and DNA methylation [24] did not find any associations between any of the four CpG probes analysed (cg27146050, cg22891070, cg16672562 and cg26749414) in fibroblasts, T cells and a lymphoblastoid cell line. However, differential *HIF3A*.*2* methylation may influence a specific subset of *HIF3A* isoforms rather than overall gene expression.

### A role in pre-eclampsia?

To our knowledge, this is the first study to report evidence for a link between pre-eclampsia and *HIF3A* methylation (Additional file 1). Pre-eclampsia is characterised by inappropriate spiral artery development during placentation [25], often in association with the development of a hypoxic environment for the developing foetus. As such, the differential *HIF3A*.*2* methylation we have observed may reflect an adaptation or response of the hypoxia response pathway to a suboptimal environment. Though the potential role of *HIF3A* in pre-eclampsia has not yet been elucidated, it has been established that the broader HIF family is involved with high levels of *HIF1A* expression in the placenta [26], and it has been proposed that dysregulated *HIF1A* may contribute to the genesis of pre-eclampsia through altered placental development.

### Impact of gestational diabetes

Previous studies have reported an association between GDM and *HIF3A* methylation [22], though this was in the area covered by *HIF3A*.*1*. While we have found novel evidence for GDM influencing average methylation in *HIF3A*.*2*, no such association was observed for *HIF3A*.*1*. However, there were relatively few women in our population-derived cohort with GDM (*n* = 17 for the *HIF3A*.*1* sub-cohort) compared to the previous study that was enriched for mothers with GDM (*n* = 68) [22]. As such, we were powered for identifying larger effects in *HIF3A*.*1* (80% power for a 3.37% increase in methylation) than the small magnitude of association previously reported (a 1.3% increase in univariate analysis, for which we had 19.2% power). *HIF3A* plays a role in glucose metabolism [27] and induces adipocyte-related gene expression [28] so may be involved in mediating the effects of maternal GDM on the infant’s development.

### Impact of offspring sex

Previous studies have considered infant sex as a covariate to control for in multivariable analysis (Additional file 1) but have focussed primarily on CpG sites within *HIF3A*.*1*. None have found an effect of sex on methylation. This is consistent with the modest association observed between infant sex and *HIF3A*.*1* methylation in this study. Interestingly however, we found a strong association between male sex and lower *HIF3A*.*2* methylation. There is little evidence in the existing literature for sex-specific differences in the function of *HIF3A* in adulthood; however, a mouse study reported that *HIF3A* expression increased in the placenta in response to maternal stress during pregnancy for placentas of male offspring but not females [29]. More broadly, there is well-established evidence for sexual dimorphism of the placenta, with reported differences in gene expression, adaptation to in utero exposures and pregnancy outcomes between the sexes [30, 31]. Sexual dimorphism may be driving the average *HIF3A*.*2* methylation differences between sexes we have observed in this study through the differential placental regulation of growth and availability of oxygen.

### Growth and adiposity

We did not find strong evidence of an association between infant adiposity or weight with average *HIF3A*.*1* methylation. Previous studies have primarily investigated methylation of three CpG sites in *HIF3A*.*1*, CpG1.2, CpG5 and CpG 6.7.8, and reported associations between methylation and adiposity or BMI in later childhood and adult timepoints [4, 6, 8, 9, 20, 21], particularly in adipose tissue but also whole blood. These studies have all used BMI as a measure of adiposity, often in combination with other measures, but BMI is not a suitable measure for adiposity at birth and instead triceps and subscapular skinfold thickness was used for our study. A positive association between methylation of these three key CpG sites and subscapular skinfold thickness has been reported in umbilical cord tissue in a previous birth cohort study [17], but we did not find evidence for associations between methylation of these three key CpG sites and growth or adiposity in site-specific analysis in cord blood (Additional file 7), potentially indicating a tissue-specific effect. A similar lack of evidence for associations was previously reported by Richmond et al. using comparable anthropometry and cord blood (*n* = 1018) [19]. However, contrasting with this previous study, which also considered BMI on a continuous scale, we did not find evidence of maternal pre-pregnancy BMI associating with average *HIF3A*.*2* methylation (*n* = 518). The CpG site previously found to be most strongly associated with pre-pregnancy BMI was *HIF3A*.*2* CpG13, but we found no evidence of an association between maternal pre-pregnancy BMI and methylation of CpG13 (Additional file 5).

### Impact of offspring *HIF3A* genotype

The impact of genetic variation on DNA methylation is pervasive [24, 32–38] but only partly understood [39]. In the current study, we captured genetic variation in an 83-kb region of *HIF3A* and upstream and downstream SNPs. There were 291 common (minor allele frequency > 0.01) SNPs in this region, none of which were in the same location as CpG sites we measured, though there were four of these SNPs within *HIF3A*.*1* or *HIF3A*.*2*. Previous studies have reported associations of rs8102595 and rs3826795 with methylation of CpG sites within *HIF3A*.1 in adult tissues [6, 8] and umbilical cord tissue [17], and we found modest evidence for these SNPs influencing average *HIF3A*.*1* methylation, though rs3826795 was more strongly associated with average *HIF3A*.*2* methylation. Of the 14 tag SNPs considered in this analysis, rs3810298 showed the greatest effect on average *HIF3A*.*2* methylation (Table 3) and is situated 6488 bp upstream from the start of *HIF3A*.*2* (Additional file 8). The other two SNPs that showed a strong association with average *HIF3A*.*2* methylation, rs112087991 and rs3826795, had a high level of linkage disequilibrium with rs3810298 (Additional file 3) indicating there may be a single SNP driving this effect on average methylation in *HIF3A*.*2*. The rs3826795 SNP was used as a tag for a total of 14 common SNPs, and of these, three (rs2072491, rs4802306 and rs3810298) are located in the second intron of *HIF3A* and positioned in regions of open chromatin that have been identified in multiple blood cell lines as part of the ENCODE project [40]. As such, the genotype of these SNPs may impact protein binding in these regions, and subsequent regulation of *HIF3A*, but this has yet to be tested. To our knowledge, we are the first to report associations between *HIF3A* genetic variation and infant cord blood methylation within the *HIF3A*.*2* region.

The key strength of this study is the combination of pregnancy health measures, infant anthropometry, genetic variation data and relatively large sample size in a population-based cohort. We have also measured locus-specific methylation allowing us to look at average methylation across two *HIF3A* regions as well as key CpG sites. The key limitation is the missing data across covariates and CpG units. As such our sample size for some analyses is smaller than some previous studies, particularly for the *HIF3A*.*1* region, limiting our power to detect small effect sizes (Additional file 1). We did not correct for multiple testing across the ten maternal and infant measures considered in association with *HIF3A*.*1* and *HIF3A*.*2* methylation. Thus, there is a possibility of false positive associations within our findings. However, these regions and measures were not agnostically chosen but based on prior literature. Further, these associations show strong evidence of persisting in multivariable linear regression modelling and were largely unchanged by the various sensitivity analyses performed. We lack information regarding participant interventions to treat pre-eclampsia or GDM that may impact findings. The functional consequences of the observed methylation variation at a specific promoter of *HIF3A* remain unclear, particularly given the lack of publicly available data on the expression and regulation of the different isoforms of this gene. Previous studies have examined total expression only, rather than isoform-specific expression, or have focussed solely on as on methylation at one of two *HIF3A* promoters that likely regulate these different isoforms [6, 9]. Future functional studies are clearly warranted in this regard to test the influence of methylation on the regulation of different isoforms of this gene. An additional consideration is that we have measured methylation in cord blood, and due to the tissue-specific nature of DNA methylation, the importance of the differential methylation we observed in this study on other tissues relevant to *HIF3A* is unclear, particularly as *HIF3A* transcription variants are differentially expressed between tissues [41].

## Conclusions

Several early-life and genetic factors appear to be associated with differential cord blood *HIF3A* DNA methylation at birth, though the potential impact of altered *HIF3A* methylation on gene expression, health and development from early childhood has yet to be well-characterised. Given the association between GDM and pre-eclampsia and both adverse maternal and infant health outcomes in later life, further studies are required to investigate the persistence of *HIF3A* methylation patterns beyond early infancy and their relevance to subsequent health outcomes.

## Methods

### Participant recruitment and follow-up

The Barwon Infant Study [42] (BIS) is an Australian birth cohort consisting of 1074 mother-infant dyads, with the aim of investigating early-life development and disease across several domains, including immune development, cardiovascular health, neurodevelopment and respiratory health. Women between 15 and 32 completed weeks of pregnancy were recruited from two hospitals but were excluded if they (1) were no longer residents in the Barwon region at the time of their child’s birth, (2) were younger than 18 years at 28 weeks of pregnancy, (3) were without Australian citizenship or permanent residency to allow for follow-up, (4) were unable to complete questionnaires or provide informed consent, (5) had a previous child in the BIS cohort (excluding twins) or (6) were planning to store their child’s cord blood privately. Neonates were excluded if they (1) had a gestational age less than 32 weeks, (2) were diagnosed with a serious illness or (3) had a genetic disease or congenital malformation. Over the 3-year recruitment period, 3933 women were contacted for recruitment and 1158 were recruited during pregnancy. After birth, 1074 mother-infant dyads remained eligible.

### Prenatal and infant measures

Health information was obtained from medical records and standardised clinical information. Gestational diabetes was defined as plasma glucose greater than 5.1 mmol/L for fasting or greater than 8.5 mmol/L 2 h after 75-g oral glucose load [43]. Pre-eclampsia was defined as per International Society for the Study of Hypertension in Pregnancy (ISSHP) criteria, with onset of high blood pressure (> 140/90) and proteinuria (> 0.3 g/24 hr) after 20 weeks of gestation [44]. Maternal pre-pregnancy BMI was calculated from self-reported weight and height measures.

Infant sex, gestational age, birth weight and anthropometry were obtained within two days of birth. *Z*-scores adjusted for sex and age were calculated for birth weight based on the revised British UK-WHO growth charts [45]**.** Triceps and subscapular skinfold thickness were measured using Holtain callipers. Sum of skinfolds (triceps and subscapular) was used as a proxy measure for central adiposity. The coefficient of reliability for newborn skinfold measures range from 75 to 93% [46].

### DNA extraction and *HIF3A* methylation

Genomic DNA was extracted from whole cord blood using the QIAamp DNA QIAcube HT Kit (QIAGEN, Hilden, Germany), following manufacturer’s instructions. Bisulphite conversion was performed using the MagPrep Lightning Conversion Kit (Zymo Research, Irvine, CA, USA). Assays for methylation of two promoter regions of *HIF3A* (*HIF3A*.*1* and *HIF3A*.*2*; hg38:chr19:46,298,243–46,298,580 and hg38:chr19:46,303,864–46,304,196, respectively) were designed using EpiDesigner (Agena Bioscience, San Diego, CA, USA). Details are shown in Additional file 8 and Additional file 9. DNA methylation and quality control were quantified as previously described [47, 48], using the SEQUENOM MassARRAY EpiTYPER platform. Methylation level was determined as the average proportion of methylation within CpG units, with each CpG unit containing one to four CpG sites. The *HIF3A*.*1* assay covered sites previously linked to adult and childhood BMI and adiposity [4, 7–9, 17–21] and measured six CpG units containing a total of 11 CpG sites. The *HIF3A*.*2* assay covered a site recently associated with maternal BMI [19] and measured 13 CpG units containing a total of 20 CpG sites. *HIF3A*.*1* was measured in a sub-sample of 490 infants, with complete methylation data for all six CpG units available for 423 infants. *HIF3A*.*2* methylation was subsequently measured in the full cohort of 938 infants, with complete methylation data for all 13 CpG units available for 609 infants.

To account for potential contribution from the cellular heterogeneity of the cord blood samples, populations of granulocytes, monocytes and lymphocytes were assessed by flow cytometry (FACsCalibur) and presented as a percentage of total white blood cells (*n* = 938) [49]. This was used in sensitivity analyses. In addition, proportions of naïve and activated T_reg_ cells (as a percentage of total CD4+ T-cells) were available for a subset of infants (*n* = 464) and were also considered in sensitivity analyses.

### *HIF3A* genotyping

Genotypes were measured using the Infinium Global Screening Array-24 v1.0 BeadChip (Illumina, San Diego, CA, USA). The Sanger Imputation Service (Wellcome Sanger Institute, Hinxton, UK) was used for imputing SNPs not captured in the initial genotyping using the EAGLE2+PBWT phasing and imputation pipeline with the Haplotype Reference Consortium reference panel [50]. The imputed SNPs were filtered for an information score greater than 0.8. The common SNPs (minor allele frequency of at least 0.01) were extracted for analysis (hg38: chr19:46,278,743–46,361,743), resulting in 291 SNPs. Due to the large number of SNPs, Haploview (Broad Institute, Cambridge, MA, USA) [51] was used to identify 14 tag SNPs using a *r*^2^ threshold of 0.1 and mandating the inclusion of two SNPs of interest identified previously [17] (rs8102595 and rs3826795).

### Statistical analysis

As methylation within a region was generally highly correlated, we considered the average methylation. Covariates of interest were identified with univariate statistical testing (unpaired two-tailed Student’s *t* test, correlation coefficient test or ANOVA as appropriate) of the average methylation for each region. The degree of correlation of tag SNPs associated with methylation was used to identify a single SNP as a proxy for the cluster of linked SNPs (based on Chi-squared tests) (Additional file 3). Linear regression models with methylation as the outcome were first built with by identifying factors associated with methylation, and then once the core model was determined, possible interaction effects between infant sex, genetic variation, and other measures on methylation were added to the model and evaluated sequentially. Participants were only included in multivariable models if they had complete data for all included variables. Subsequent analysis of specific CpG unit methylation was performed to identify secondary maternal or infant measures to be included in sensitivity analysis. Other covariates added separately to the linear regression model for sensitivity analysis included proportions of various white blood cells in the cord blood samples and technical variables (bisulphite conversion batch and methylation quantification batch). Analyses including blood cell proportions were also adjusted for infant exposure to labour prior to delivery (any or none). The covariates examined in sensitivity analysis were considered to impact on the model if they altered the beta value of the main effects by 10% or more. The likelihood ratio test was used to compare nested linear regression models to evaluate if additional terms in the model were useful for explaining additional outcome variance. Stata 15 IC (StataCorp, College Station, TX, USA) was used for analysis.

## Additional files

Additional file 1: Summary table of studies investigating *HIF3A* DNA methylation in the context of obesity, adiposity, or early-life influences. (DOCX 21 kb)

Additional file 2: Tables of the pairwise associations between the *HIF3A*.*1* and *HIF3A*.*2* CpG units measured in this study. The first spreadsheet in the file gives the *r* values, the second spreadsheet gives the *p* values. (XLSX 18 kb)

Additional file 3: Visualisation of linkage disequilibrium between the 14 tag SNPs considered in this analysis from Haploview. The numbers in individual pairwise boxes are the D’, a measure of linkage disequilibrium. A stronger red colour for a box indicates the two SNPs are more strongly linked. The white bar at the top indicates the relative genomic position of each SNP. (DOCX 50 kb)

Additional file 4: Summary tables of the univariate tests between rs3810298 genotype and (a) pre-eclampsia, (b) gestational diabetes, (c) birth weight, and (d) gestational age. (XLSX 11 kb)

Additional file 5: Table of associations between cohort characteristics and methylation of unit-specific *HIF3A*.*2* CpG methylation. (DOCX 23 kb)

Additional file 6: Table of final linear regression model adjusting for all key variables with unit-specific *HIF3A*.*2* methylation as outcome, applied to all measured *HIF3A*.*2* CpG units. (DOCX 20 kb)

Additional file 7: Table of associations between cohort characteristics and methylation of unit-specific *HIF3A*.*1* CpG methylation. (DOCX 18 kb)

Additional file 8: Annotated UCSC genome browser (http://genome.ucsc.edu) view of the HIF3A gene region and Epityper assays. (a) *HIF3A* gene on chromosome 19. Gene transcription is from left to right. Multiple splice variants are shown, with solid dark segments indicating exons, and the connecting lines indicating introns. The positions of SNPs included in this analysis are shown in green and labelled. (b) The *HIF3A*.*1* region, with CpG sites in red. (c) The *HIF3A*.*2* region, with CpG sites in red. The measurable CpG sites are numbered based on the predicted cleavage pattern from the Epityper in silico prediction. CpG units that contain CpG sites of interest from previous literature have the CpG site reference in brackets beneath the number. (DOCX 111 kb)

Additional file 9: Table of primer and assay information for *HIF3A*.*1* and *HIF3A*.*2*. (DOCX 13 kb)

## Data Availability

The datasets used and/or analysed during the current study are available from the corresponding author on reasonable request.

## Abbreviations

A: Adenine
ANOVA: Analysis of variance
BIS: Barwon Infant Study
BMI: Body mass index
C: Cytosine
CI: Confidence interval
CpG: Cytosine-guanine dinucleotide
DNA: Deoxyribonucleic acid
DOHaD: Developmental Origins of Health and Disease
EWAS: Epigenome-wide association study
G: Guanine
GDM: Gestational diabetes mellitus
hg38: Genome Reference Consortium human genome build 38
*HIF3A*: Hypoxia-inducible factor 3α gene
HIF-3α: Hypoxia-inducible factor 3α
ISSHP: International Society for the Study of Hypertension in Pregnancy
PBWT: Positional Burrows-Wheeler transform
SD: Standard deviation
SE: Standard error
SNP: Single nucleotide polymorphism
T: Thymine
UK-WHO: United Kingdom-World Health Organisation
