# Supplementary material for: Early-life determinants of hypoxia-inducible factor 3A gene (HIF3A) methylation: a birth cohort study
Source: Clin Epigenetics. 2019 Jul 1;11:96. doi: 10.1186/s13148-019-0687-0 (PMC6604333; doi:10.1186/s13148-019-0687-0)
Supplement: Supplementary file 1 — Summary table of studies investigating HIF3A DNA methylation in the context of obesity, adiposity, or early-life influences. (DOCX 21 kb) [file 13148_2019_687_MOESM1_ESM.docx]

| **Additional file 1***.* Summary of studies investigating *HIF3A* DNA methylation in the context of obesity, adiposity, or early life influences. | | | | | | | | |
| --- | --- | --- | --- | --- | --- | --- | --- | --- |
| **Author** | **Year** | **Time** | **Tissue** | **Cohort** | **CpGs** | **SNPs** | **Exposure/Outcome** | ***HIF3A* Findings** |
| Dick et al. | 2014 | Adult | Whole blood. Skin and adipose in MuTHER replication cohort | European ancestry in the Cardiogenics Consortium, n=459.  Replication cohorts were MARTHA (n=339), KORA (n=1789), and MuTHER (women, n=635 for adipose and n=395 for skin) | 450k array (inc. cg27146050  cg16672562  cg22891070). | rs8102595  rs3826795  (D’=1)  Considered other SNPS within 1Mb | BMI (log transformed). Key covariates were age, sex, smoking, methylation array batch and centre. | The three *HIF3A* CpGs were strongly associated with BMI in whole blood (3.2-7.8% increase in BMI per 10% methyl increase in discovery cohort) and in adipose (6.2-11.9% increase in BMI per 10% methyl. increase).  In adipose, *HIF3A* methylation and gene expression were weakly associated.  The two key SNPs associated with cg22891070 in all three tissue types.  SNPs did not associate with BMI/adiposity, but analysis had low power. |
| Pan et al. | 2015 | Birth | Umbilical | GUSTO, n=991 | cg27146050  cg16672562  cg22891070. | rs8102595  rs3826795 | Birth weight, length, BMI, and subscapular and triceps skinfold thicknesses (and ratio) | Weight (2.05-3.61g per 10% methyl. increase) and BMI (1.35-2.40g/cm^2^ per 10% methyl. increase) associated with all 3 CpGs, subscapular (3.27-5.44mm per 10% methyl. increase) and skinfold ratio (3.02-4.67 increase per 10% methyl. increase) associated with cg16672562 and cg22891070.  Both SNPS associated with methyl. at all three CpGs, adjusting for SNPs did not alter findings. Interaction effect between birthweight and rs3826795 on methylation. |
| Demerath et al. | 2015 | Adult  (47 – 70 years old in ARIC) | Leukocyte.  Replication cohorts used whole blood, CD4+ T cells, and adipose tissue | ARIC (African American), n=2097.  The 3 replication cohorts were FHS (white), n=2377; GOLDN (white), n=991; and MTHER (women), n=648 | 450k array (inc. cg27146050  cg16672562  cg22891070). | SNPs in 500kb windows around the 37 replicated BMI CpGs | BMI, waist circ., BMI change.  Key covariates included sex, age, smoking status, and genetic ancestry. | 37 CpGs associated with BMI and replicated, 8 associated with waist circ. and replicated.  19 BMI CpGs also associated with diabetes, 18 BMI and 1 waist circ. also in adipose tissue.  SNPs reportedly unlikely to confound methyl. associations.  cg27146050, cg16672562, cg22891070 positively associated with BMI in ARIC but not GOLDN and FHS. cg16672562 also associated with BMI change. |
| Agha et al. | 2015 | Adult (mean age: 47 years) | Whole blood  Adipose | New England Family Study (n=106) | 450k array (inc. cg27146050  cg16672562  cg22891070), *HIF3A* considered as candidate gene. | None | BMI, android fat mass, android/gynoid fat ratio, trunk/limb fat ratio. Key covariates were race, sex and smoking status | Adipose tissue methylation profile associated with all four adiposity measures, blood methylation was not associated with any of the four measures.  Methylation at the three *HIF3A* probes was associated with BMI in adipose tissue (0.3-0.6% methy. increase per kg/m^2^) but not blood. |
| Huang et al. | 2015 | Adult  (NHS: 30 – 55 yo. HPFS: 40 – 75 yo.) | Whole blood | NHS (women, n=8109) and HPFS (men, n=6761), all participants of European ancestry | Not measured directly, but implicitly cg22891070. | rs8102595  rs3826795 | Several B vitamins, consumption of ‘junk’ food subtypes, physical activity, change in weight/BMI over 10 years. | rs3826795 not associated with adiposity.  Vitamin B intake positively associated with change in BMI.  Vit. B and folate interaction with genetic association for change in BMI.  Vitamin B not associated with methylation. |
| Rönn et al. | 2015 | Adult (mean age: 32.4 years, SD: 12.8 years) | Subcutaneous adipose tissue | n=190 from Sweden and Denmark, made up of male (n=96) and female (n=94) cohorts. | 450k array (inc. cg27146050  cg16672562  cg22891070) | None | BMI, age, HbA1c, genome-wide gene expression. | Methylation of 7 *HIF3A* CpG sites associated with BMI in female cohort, 2 different sites associated with BMI in males.  Methylation of 2 *HIF3A* CpG sites associated with expression of several (4 for one, 9 for the mRNAs. |
| Richmond et al. | 2016 | Longitudinally across birth, childhood, adolescence | Cord blood and whole blood | ALSPAC, n=974 | 450k array (inc. cg27146050  cg16672562  cg22891070, also cg20667364, cg26749414, cg25196389, cg23548163). | rs8102595  rs3826795 | BMI, fat mass, lean mass, maternal prepregnancy BMI  Main covariates were age, sex, smoking status and bisulphite batch | cg27146050 associated with adolescent BMI in cross-sectional analysis (4.7% increase in BMI per 10% methyl increase in discovery cohort), not other CpGs or timepoints.  Positive correlation between earlier BMI and later methylation in longitudinal analysis, particularly for cg27146050 (1% increase in child methyl. per 1kg birthweight increase, 0.3% increase in adolescent methyl. per 10% child BMI increase).  Maternal prepreg. BMI negative correlated with cg27146050, more strongly and positively correlated with cg20667364, cg26749414, cg25196389, and cg23548163 (largest effect: ~2.6% increase in birth methyl. per 10% maternal BMI increase), only at the birth timepoint, and cg27146050 association reversed by adolescence. |
| Huang et al. | 2016 | Adult | Blood and subcutaneous adipose tissue | n=143 from LEAP Project in United States. | 450k array (inc. cg27146050  cg16672562  cg22891070). Methylation considered both on a CpG site level and a gene level. | None | BMI, race, gender, with key covariates including age, smoking, and socioeconomic index. | Average *HIF3A* methylation correlated across blood and adipose tissue (r=0.40).  *HIF3A* methylation in both adipose and blood positively associated with BMI, with the association stronger in adipose. |
| Wang et al. | 2016 | Childhood, aged 7-17 years | Whole blood | n=220 from Chinese CPOOA cohort (110 pairs of severely obese cases and age- and sex-matched controls) | A locus of *HIF3A*, measured with EpiTYPER (the same region as *HIF3A.1* in our study), which covered cg27146050  cg16672562  cg22891070. | None | BMI and associated anthropometry, biochemicals in blood (including cholesterols, alanine aminotransferase, glucose and insulin). | Obese group had 2.3-2.9% increased methylation at cg22891070 and one other CpG unit (CpG5 and CpG11 in our study).  After adjustment for BMI, alanine aminotransferase was the only biochemical associated with methylation (CpG11, r=0.23). |
| Main et al. | 2016 | Adult, aged 32-83 years | Whole blood, subcutaneous adipose. Gene expression in adipose and muscle. | n=137 from 48 families in Danish EUGENE2 cohort. All participants were direct relatives of T2D patients. | A locus of *HIF3A*, measured with EpiTYPER, covering 4 CpG sites including cg16672562  cg22891070. | None | BMI, *HIF3A* gene expression, T2D status. Adjusted for age, sex, and haemoglobin A1c level. | cg16672562 methylation in blood correlated with cg16672562 methylation in adipose (r=0.35), but other CpG sites not correlated across tissues.  cg22891070 blood methylation associated with BMI (0.3% methyl. increase per kg/m^2^ increase).  Adipose methylation was not associated with BMI.  Adipose *HIF3A* expression negatively associated with BMI (-2.9% expression per kg/m^2^ increase), but not associated with adipose methylation. |
| Pfeiffer et al. | 2016 | Adult (mean age: 50 years, SD: 14 years) | Paired subcutaneous adipose tissue (SAT) and visceral adipose tissue (VAT) | n=87 for methylation, n=603 for gene expression. | 11 CpG sites within intron 1 of *HIF3A*, including cg27146050  cg16672562  cg22891070 (pyrosequencing). | rs8102595  rs3826795 | BMI and associated anthropometry, biochemicals in blood (including cholesterols), T2D status, *HIF3A* and *LEP* gene expression. | Methylation of cg22891070 in VAT positively associated with VAT (r=0.47) and SAT (r=0.65) mass.  Higher methylation across CpG sites in obese compared to non-obese individuals, but non-significant.  rs8102595 minor allele associated with increased *HIF3A* methylation, but not mRNA expression. |
| Haertle et al. | 2017 | Birth | Cord blood | n=132 (44 I-GDM, 24 D-GDM and 64 controls) | 450k array, with pyrosequencing of key candidate genes (inc. *HIF3A*, covering cg27146050  cg16672562  cg22891070). | None | GDM (I- and D-GDM). Main covariates were mat. preg. BMI, gestational week and sex. | 1564 CpG sites genome-wide associated with differential methylation between I-GDM and control pregnancies, no significance with D-GDM.  In pyrosequencing, *HIF3A* mean methylation across the 11 sequenced CpGs was positively associated with GDM (1.3% methy. increase, 2.3% increase in adjusted model), with 4 specific CpGs associated more strongly (1.7-3.7% increase in adjusted model). |
| Lee et al. | 2017 | Childhood, aged 12-15 years (mean age: 13.9 years) | Whole blood | n=692 (305 obese cases and 387 controls, Korean population) | cg27146050, cg46801562, cg22891070, cg16672562 and cg46801675  (pyrosequencing). | 14 SNPs from *HIF3A* exome sequencing | BMI, hip-waist (H-W) ratio, fasting glucose level. Age and sex considered as covariates. | cg46801562 was associated with obesity group (3.1% methyl. increase).  cg16672562 positively associated with all three main measures in adjusted model (0.3% methyl. increase per kg/m^2^ increase, 0.003% per H-W ratio unit, 0.06% per mg/dL), and cg16672562 methylation was independent of genetic variation.  All CpGs were associated with fasting glucose levels (effect of -0.04 to 0.07% change to methyl. per mg/dL increase). |
| 450k = Infinium Human Methylation450 BeadChip, ALSPAC = Avon Longitudinal Study of Parents and Children, ARIC = Atherosclerosis Risk in Communities study, BMI = body-mass index, CPOOA = Comprehensive Prevention project for Overweight and Obese Adolescents, EUGENE2 = European Network on Functional Genomics of Type 2 Diabetes, FHS = Framingham Heart Study, GDM (I- and D-) = gestational diabetes mellitus (insulin-treated and diet-treated) , GOLDN = Genetics of Lipid Lowering Drugs and Diet Network Study, GUSTO = Growing Up in Singapore Towards Healthy Outcomes, HPFS = Health Professionals Follow-Up Study, KORA = Cooperative Health Research in the Region of Augsburg, LEAP = Longitudinal Effects on Aging Perinatal, MARTHA = MARseille THrombosis Association, MuTHER = Multiple Tissue Human Expression Resource, NHS = Nurses’ Health Study, SD = standard deviation, SNP = single nucleotide polymorphism, T2D = type 2 diabetes. | | | | | | | | |
