# Supplementary material for: Early-life determinants of hypoxia-inducible factor 3A gene (HIF3A) methylation: a birth cohort study
Source: Clin Epigenetics. 2019 Jul 1;11:96. doi: 10.1186/s13148-019-0687-0 (PMC6604333; doi:10.1186/s13148-019-0687-0)
Supplement: Supplementary file 3 — Visualisation of linkage disequilibrium between the 14 tag SNPs considered in this analysis from Haploview. The numbers in individual pairwise boxes are the D’, a measure of linkage disequilibrium. A stronger red colour for a box indicates the two SNPs are more strongly linked. The white bar at the top indicates the relative genomic position of each SNP. (DOCX 50 kb) [file 13148_2019_687_MOESM3_ESM.docx]

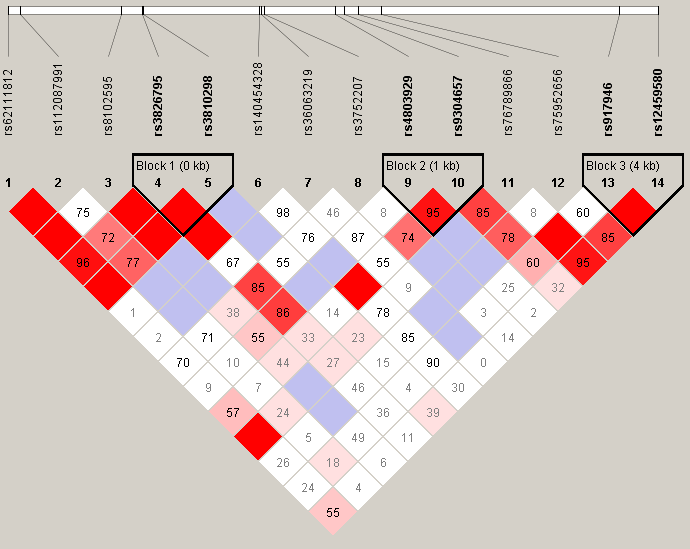


**Additional file 3.** Visualisation of linkage disequilibrium between the 14 tag SNPs considered in this analysis from Haploview. The numbers in individual pairwise boxes are the D’, a measure of linkage disequilibrium. A stronger red colour for a box indicates the two SNPs are more strongly linked. The white bar at the top indicates the relative genomic position of each SNP.
