# Supplementary material for: Early-life determinants of hypoxia-inducible factor 3A gene (HIF3A) methylation: a birth cohort study
Source: Clin Epigenetics. 2019 Jul 1;11:96. doi: 10.1186/s13148-019-0687-0 (PMC6604333; doi:10.1186/s13148-019-0687-0)
Supplement: Supplementary file 5 — Table of associations between cohort characteristics and methylation of unit-specific HIF3A.2 CpG methylation. (DOCX 23 kb) [file 13148_2019_687_MOESM5_ESM.docx]

| Additional file 5. Associations between cohort characteristics and methylation of specific *HIF3A.2* CpG unit methylation. | | | | | | | | | | | | | | |
| --- | --- | --- | --- | --- | --- | --- | --- | --- | --- | --- | --- | --- | --- | --- |
|  | **CpG1 (n=920)** | | **CpG4 (n=782)** | | **CpG5.6.7 (n=875)** | | **CpG8.9 (n=911)** | | **CpG10 (n=863)** | | **CpG11 (n=847)** | | **CpG12 (n=814)** | |
| Maternal | **r** | **p** | **r** | **p** | **r** | **p** | **r** | **p** | **r** | **p** | **r** | **p** | **r** | **p** |
| Age (years) | -0.03 | 0.34 | -0.02 | 0.64 | -0.04 | 0.21 | -0.04 | 0.24 | -0.04 | 0.30 | <0.01 | 0.99 | 0.02 | 0.50 |
| Pre-pregnancy BMI (kg/m^2^) | 0.02 | 0.53 | 0.06 | 0.10 | 0.03 | 0.46 | 0.03 | 0.48 | 0.06 | 0.12 | 0.08 | 0.04 | 0.01 | 0.81 |
|  | **Effect (SE)** | **p** | **Effect (SE)** | **p** | **Effect (SE)** | **p** | **Effect (SE)** | **p** | **Effect (SE)** | **p** | **Effect (SE)** | **p** | **Effect (SE)** | **p** |
| Smoking | 1.16 (0.62) | 0.06 | 2.07 (1.20) | 0.08 | 1.34 (1.00) | 0.18 | 1.34 (0.98) | 0.17 | 1.77 (1.49) | 0.24 | 0.93 (1.14) | 0.41 | 0.76 (1.26) | 0.55 |
| Gestational diabetes | 1.84 (1.13) | 0.10 | 4.74 (2.16) | 0.03 | 3.18 (1.83) | 0.08 | 3.51 (1.81) | 0.05 | 6.58 (2.72) | 0.02 | 1.96 (2.14) | 0.36 | 4.76 (2.32) | 0.04 |
| Pre-eclampsia | -3.42 (1.34) | 0.01 | -7.88 (2.51) | <0.001 | -6.22 (2.15) | <0.001 | -6.77 (2.08) | <0.001 | -6.95 (3.28) | 0.03 | -5.20 (2.37) | 0.03 | -3.11 (2.70) | 0.25 |
|  |  |  |  |  |  |  |  |  |  |  |  |  |  |  |
| Infant | **r** | **p** | **r** | **p** | **r** | **p** | **r** | **p** | **r** | **p** | **r** | **p** | **r** | **p** |
| Gestational age (weeks) | 0.15 | <0.001 | 0.13 | <0.001 | 0.16 | <0.001 | 0.17 | <0.001 | 0.15 | <0.001 | 0.15 | <0.001 | 0.18 | <0.001 |
| Birth weight (g) | 0.03 | 0.30 | 0.01 | 0.84 | 0.04 | 0.29 | 0.05 | 0.12 | 0.05 | 0.17 | 0.03 | 0.31 | 0.07 | 0.06 |
| Z-score | -0.03 | 0.36 | -0.05 | 0.20 | -0.02 | 0.50 | -0.02 | 0.58 | -0.01 | 0.75 | -0.03 | 0.38 | -0.01 | 0.78 |
| Tricep+subscular sum (mm) | 0.04 | 0.21 | 0.01 | 0.86 | 0.00 | 0.98 | 0.01 | 0.79 | 0.01 | 0.85 | 0.03 | 0.48 | 0.02 | 0.52 |
|  | **Effect (SE)** | **p** | **Effect (SE)** | **p** | **Effect (SE)** | **p** | **Effect (SE)** | **p** | **Effect (SE)** | **p** | **Effect (SE)** | **p** | **Effect (SE)** | **p** |
| Sex (male) | -2.05 (0.45) | <0.001 | -3.78 (0.86) | <0.001 | -4.01 (0.71) | <0.001 | -3.23 (0.70) | <0.001 | -5.16 (1.07) | <0.001 | -3.67 (0.83) | <0.001 | -3.19 (0.91) | <0.001 |
|  | **CpG13 (n=870)** | | **CpG14.15.16.17 (n=899)** | | **CpG18 (n=865)** | | **CpG24 (n=861)** | | **CpG25 (n=878)** | | **CpG26.27 (n=893)** | |  |  |
| Maternal | **r** | **P** | **r** | **p** | **r** | **p** | **r** | **p** | **r** | **p** | **r** | **p** |  |  |
| Age (years) | -0.03 | 0.42 | -0.01 | 0.67 | -0.01 | 0.76 | -0.04 | 0.28 | -0.01 | 0.87 | -0.01 | 0.79 |  |  |
| Pre-pregnancy BMI (kg/m^2^) | 0.04 | 0.28 | 0.03 | 0.43 | 0.04 | 0.26 | 0.06 | 0.13 | 0.04 | 0.22 | 0.03 | 0.35 |  |  |
|  | **Effect (SE)** | **p** | **Effect (SE)** | **p** | **Effect (SE)** | **p** | **Effect (SE)** | **p** | **Effect (SE)** | **p** | **Effect (SE)** | **p** |  |  |
| Smoking | 1.83 (1.54) | 0.23 | 0.97 (1.05) | 0.36 | 1.71 (1.41) | 0.22 | 1.44 (1.33) | 0.28 | 0.07 (1.17) | 0.95 | 1.00 (1.15) | 0.22 |  |  |
| Gestational diabetes | 5.03 (2.82) | 0.08 | 3.50 (1.93) | 0.07 | 4.99 (2.52) | 0.05 | 3.56 (2.34) | 0.13 | 4.73 (2.12) | 0.03 | 4.02 (2.06) | <0.001 |  |  |
| Pre-eclampsia | -8.60 (3.18) | 0.01 | -4.36 (2.21) | 0.05 | -7.68 (2.92) | 0.01 | -7.50 (2.74) | 0.01 | -6.64 (2.57) | 0.01 | -6.10 (2.48) | 0.06 |  |  |
|  |  |  |  |  |  |  |  |  |  |  |  |  |  |  |
| Infant | **r** | **p** | **r** | **p** | **r** | **p** | **r** | **p** | **r** | **p** | **r** | **p** |  |  |
| Gestational age (weeks) | 0.17 | <0.001 | 0.17 | <0.001 | 0.17 | <0.001 | 0.20 | <0.001 | 0.13 | <0.001 | 0.18 | <0.001 |  |  |
| Birth weight (g) | 0.06 | 0.06 | 0.04 | 0.20 | 0.04 | 0.20 | 0.09 | 0.01 | 0.06 | 0.06 | 0.05 | 0.11 |  |  |
| Z-score | <0.01 | 0.94 | -0.03 | 0.38 | -0.03 | 0.34 | 0.01 | 0.77 | 0.02 | 0.54 | -0.03 | 0.45 |  |  |
| Tricep+subscular sum (mm) | <0.01 | 0.98 | 0.01 | 0.71 | 0.02 | 0.60 | 0.00 | 0.90 | 0.04 | 0.28 | 0.01 | 0.79 |  |  |
|  | **Effect (SE)** | **p** | **Effect (SE)** | **p** | **Effect (SE)** | **p** | **Effect (SE)** | **p** | **Effect (SE)** | **p** | **Effect (SE)** | **p** |  |  |
| Sex (male) | -5.49 (1.09) | <0.001 | -3.66 (0.75) | <0.001 | -4.58 (1.01) | <0.001 | -4.28 (0.95) | <0.001 | -3.75 (0.83) | <0.001 | -3.92 (0.82) | 0.54 |  |  |
