# Supplementary material for: Early-life determinants of hypoxia-inducible factor 3A gene (HIF3A) methylation: a birth cohort study
Source: Clin Epigenetics. 2019 Jul 1;11:96. doi: 10.1186/s13148-019-0687-0 (PMC6604333; doi:10.1186/s13148-019-0687-0)
Supplement: Supplementary file 6 — Table of final linear regression model adjusting for all key variables with unit-specific HIF3A.2 methylation as outcome, applied to all measured HIF3A.2 CpG units. (DOCX 20 kb) [file 13148_2019_687_MOESM6_ESM.docx]

| Additional file 6. Final linear regression model adjusting for all key variables with unit-specific *HIF3A.*2 methylation as outcome, applied to all measured *HIF3A.*2 CpG units. | | | | | | | | | | | | | | |
| --- | --- | --- | --- | --- | --- | --- | --- | --- | --- | --- | --- | --- | --- | --- |
|  | **Average (n=513)** | | **CpG 1 (n=765)** | | **CpG 4 (n=653)** | | **CpG 5.6.7 (n=729)** | | **CpG 8.9 (n=758)** | | **CpG 10 (n=721)** | | **CpG 11 (n=703)** | |
|  | **β (SE)** | **p** | **β (SE)** | **p** | **β (SE)** | **p** | **β (SE)** | **p** | **β (SE)** | **p** | **β (SE)** | **p** | **β (SE)** | **p** |
| GDM | 4.62 (1.77) | 0.009 | 1.99 (1.08) | 0.07 | 5.47 (2.08) | 0.009 | 3.59 (1.73) | 0.04 | 3.90 (1.71) | 0.02 | 6.27 (2.54) | 0.01 | 2.60 (2.03) | 0.20 |
| Pre-eclampsia | -5.20 (2.19) | 0.02 | -2.98 (1.39) | 0.03 | -7.48 (2.73) | 0.006 | -5.56 (2.19) | 0.01 | -6.12 (2.12) | 0.004 | -6.03 (3.29) | 0.07 | -5.44 (2.44) | 0.03 |
| Sex (male) | -3.77 (0.76) | <0.001 | -2.06 (0.46) | <0.001 | -3.49 (0.91) | <0.001 | -3.76 (0.73) | <0.001 | -3.39 (0.71) | <0.001 | -5.07 (1.07) | <0.001 | -4.02 (0.84) | <0.001 |
| Gest. age (weeks) | 1.21 (0.27) | <0.001 | 0.62 (0.17) | <0.001 | 1.13 (0.33) | 0.001 | 1.12 (0.27) | <0.001 | 1.21 (026) | <0.001 | 1.68 (0.39) | <0.001 | 1.11 (0.31) | <0.001 |
| rs3810298 |  |  |  |  |  |  |  |  |  |  |  |  |  |  |
| C/T | -8.31 (0.97) | <0.001 | -4.83 (0.61) | <0.001 | -5.86 (1.18) | <0.001 | -7.15 (0.96) | <0.001 | -6.78 (0.94) | <0.001 | -12.63 (1.42) | <0.001 | -8.99 (1.11) | <0.001 |
| T/T | -16.91 (2.73) | <0.001 | -8.98 (1.79) | <0.001 | -10.82 (3.52) | 0.002 | -16.89 (2.99) | <0.001 | -16.09 (2.82) | <0.001 | -24.43 (4.04) | <0.001 | -19.88 (3.12) | <0.001 |
|  | **CpG 12 (n=679)** | | **CpG 13 (n=723)** | | **CpG 14.15.16.17 (n=747)** | | **CpG 18 (n=723)** | | **CpG 24 (n=717)** | | **CpG 25 (n=732)** | | **CpG 26.27 (n=744)** | |
|  | **β (SE)** | **p** | **β (SE)** | **p** | **β (SE)** | **p** | **β (SE)** | **p** | **β (SE)** | **p** | **β (SE)** | **p** | **β (SE)** | **p** |
| GDM | 3.75 (2.19) | 0.09 | 5.66 (2.67) | 0.04 | 3.33 (1.76) | 0.06 | 4.96 (2.30) | 0.03 | 3.65 (2.22) | 0.10 | 4.23 (1.90) | 0.03 | 3.96 (1.94) | 0.04 |
| Pre-eclampsia | -2.19 (2.75) | 0.43 | -8.09 (3.27) | 0.01 | -4.19 (2.21) | 0.06 | -6.51 (2.89) | 0.03 | -6.35 (2.83) | 0.03 | -5.51 (2.44) | 0.02 | -4.74 (2.47) | 0.06 |
| Sex (male) | -2.83 (0.92) | 0.002 | -5.39 (1.11) | <0.001 | -3.84 (0.74) | <0.001 | -5.03 (0.98) | <0.001 | -4.14 (0.97) | <0.001 | -3.40 (0.81) | <0.001 | -4.18 (0.83) | <0.001 |
| Gest. age (weeks) | 1.51 (0.34) | <0.001 | 1.72 (0.40) | <0.001 | 1.29 (0.27) | <0.001 | 1.76 (0.36) | <0.001 | 1.63 (0.35) | <0.001 | 1.09 (0.29) | <0.001 | 1.51 (0.30) | <0.001 |
| rs3810298 |  |  |  |  |  |  |  |  |  |  |  |  |  |  |
| C/T | -10.13 (1.23) | <0.001 | -11.36 (1.45) | <0.001 | -10.01 (0.98) | <0.001 | -13.12 (1.29) | <0.001 | -9.54 (1.28) | <0.001 | -12.89 (1.06) | <0.001 | -8.28 (1.10) | <0.001 |
| T/T | -17.25 (3.37) | <0.001 | -24.83 (4.35) | <0.001 | -18.00 (2.83) | <0.001 | -24.02 (3.70) | <0.001 | -21.70 (3.76) | <0.001 | -25.70 (3.17) | <0.001 | -17.54 (3.29) | <0.001 |
| Effect sizes (β) given as percentage methylation. β for rs3810298 categories are difference from homozygote major allele (C/C). GDM = gestational diabetes, SE = standard error. | | | | | | | | | | | | | | |
