# Supplementary material for: Early-life determinants of hypoxia-inducible factor 3A gene (HIF3A) methylation: a birth cohort study
Source: Clin Epigenetics. 2019 Jul 1;11:96. doi: 10.1186/s13148-019-0687-0 (PMC6604333; doi:10.1186/s13148-019-0687-0)
Supplement: Supplementary file 7 — Table of associations between cohort characteristics and methylation of unit-specific HIF3A.1 CpG methylation. (DOCX 18 kb) [file 13148_2019_687_MOESM7_ESM.docx]

| Additional file 7. Associations between cohort characteristics and methylation of unit-specific *HIF3A.1* CpG methylation. | | | | | | | | | | | | |
| --- | --- | --- | --- | --- | --- | --- | --- | --- | --- | --- | --- | --- |
|  | **CpG1.2 (n=430)** | | **CpG5 (n=479)** | | **CpG6.7.8 (n=486)** | | **CpG9.10 (n=488)** | | **CpG11 (n=484)** | | **CpG13.14 (n=484)** | |
| Maternal |  |  |  |  |  |  |  |  |  |  |  |  |
|  | **r** | **p** | **r** | **p** | **r** | **p** | **r** | **p** | **r** | **p** | **r** | **p** |
| Age (years) | 0.02 | 0.63 | 0.08 | 0.07 | 0.05 | 0.29 | 0.05 | 0.29 | 0.04 | 0.42 | 0.00 | 0.93 |
| Pre-pregnancy BMI (kg/m^2^) | <0.01 | 0.99 | -0.01 | 0.92 | -0.03 | 0.57 | -0.04 | 0.47 | 0.01 | 0.88 | -0.04 | 0.40 |
|  | **Effect (SE)** | **p** | **Effect (SE)** | **p** | **Effect (SE)** | **p** | **Effect (SE)** | **p** | **Effect (SE)** | **p** | **Effect (SE)** | **p** |
| Smoking | 0.48 (1.12) | 0.67 | -1.05 (1.26) | 0.40 | -0.55 (0.88) | 0.54 | -0.88 (1.00) | 0.38 | -1.02 (0.86) | 0.24 | 0.21 (1.07) | 0.84 |
| Gestational diabetes | 1.18 (2.00) | 0.55 | -1.04 (2.29) | 0.65 | -1.16 (1.63) | 0.48 | -2.23 (1.86) | 0.23 | -2.23 (1.57) | 0.16 | 0.04 (2.00) | 0.99 |
| Pre-eclampsia | -1.39 (2.10) | 0.51 | 0.47 (2.30) | 0.84 | -1.53 (1.58) | 0.33 | 0.46 (1.79) | 0.80 | -0.98 (1.54) | 0.52 | -0.21 (1.92) | 0.91 |
|  |  |  |  |  |  |  |  |  |  |  |  |  |
| Infant |  |  |  |  |  |  |  |  |  |  |  |  |
|  | **r** | **p** | **r** | **p** | **r** | **p** | **r** | **p** | **r** | **p** | **r** | **p** |
| Gestational age (weeks) | -0.07 | 0.16 | 0.12 | 0.007 | -0.03 | 0.56 | 0.01 | 0.89 | 0.02 | 0.61 | 0.03 | 0.49 |
| Birth weight (g) | -0.02 | 0.71 | 0.05 | 0.24 | 0.00 | 0.99 | -0.01 | 0.87 | 0.04 | 0.42 | 0.03 | 0.50 |
| Z-score | 0.01 | 0.88 | -0.04 | 0.40 | 0.00 | 1.00 | -0.03 | 0.53 | 0.01 | 0.85 | 0.02 | 0.68 |
| Tricep+subscular sum (mm) | -0.02 | 0.74 | -0.01 | 0.88 | 0.02 | 0.72 | 0.03 | 0.58 | 0.05 | 0.25 | 0.04 | 0.39 |
|  | **Effect (SE)** | **p** | **Effect (SE)** | **p** | **Effect (SE)** | **p** | **Effect (SE)** | **p** | **Effect (SE)** | **p** | **Effect (SE)** | **p** |
| Sex (male) | -0.81 (0.74) | 0.28 | -0.18 (0.82) | 0.83 | -0.88 (0.58) | 0.13 | -0.58 (0.66) | 0.38 | -1.27 (0.56) | 0.03 | -0.07 (0.70) | 0.93 |
