# Supplementary material for: Early-life determinants of hypoxia-inducible factor 3A gene (HIF3A) methylation: a birth cohort study
Source: Clin Epigenetics. 2019 Jul 1;11:96. doi: 10.1186/s13148-019-0687-0 (PMC6604333; doi:10.1186/s13148-019-0687-0)
Supplement: Supplementary file 8 — Annotated UCSC genome browser (http://genome.ucsc.edu) view of the HIF3A gene region and Epityper assays. (a) HIF3A gene on chromosome 19. Gene transcription is from left to right. Multiple splice variants are shown, with solid dark segments indicating exons, and the connecting lines indicating introns. The positions of SNPs included in this analysis are shown in green and labelled. (b) The HIF3A.1 region, with CpG sites in red. (c) The HIF3A.2 region, with CpG sites in red. The measurable CpG sites are numbered based on the predicted cleavage pattern from the Epityper in silico prediction. CpG units that contain CpG sites of interest from previous literature have the CpG site reference in brackets beneath the number. (DOCX 111 kb) [file 13148_2019_687_MOESM8_ESM.docx]

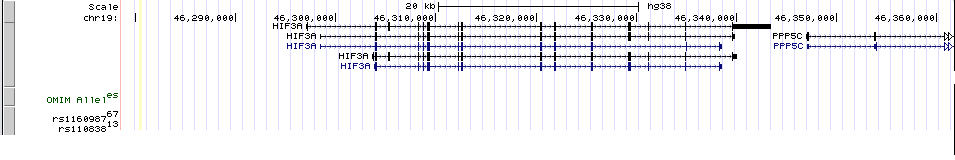


*HIF3A.1*

*HIF3A.2*

rs62111812

rs112087991

rs8102595

rs3826795

rs3810298

rs140454328

rs36063219

rs3752207

rs4803929

rs9304657

rs76789866

rs75952656

rs917946

rs12459580

**(A)**


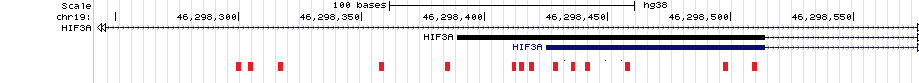


1.2

(cg27146050)

5

(cg22891070)

6.7.8

(cg16672562)

9.10

11

13.14

**(B)**


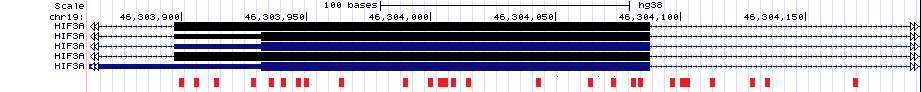


1

4

7.6.5

24

9.8

10

11

12

13

(cg26749414)

17.16.15.14

18

25

27.26

**(C)**

**Additional file 8.** UCSC genome browser [http://ucsc.genome.edu] view of the *HIF3A* gene region and Epityper assays. (a) *HIF3A* gene on chromosome 19. Gene transcription is from left to right. Multiple splice variants are shown, with solid dark segments indicating exons, and the connecting lines indicating introns. The positions of SNPs included in this analysis are shown in green and labelled. (b) The *HIF3A.1* region, with CpG sites in red. (c) The *HIF3A.2* region, with CpG sites in red. The measurable CpG sites are numbered based on the predicted cleavage pattern from the Epityper *in silico* prediction. CpG units that contain CpG sites of interest from previous literature have the CpG site reference in brackets beneath the number.
