# Supplementary material for: Early-life determinants of hypoxia-inducible factor 3A gene (HIF3A) methylation: a birth cohort study
Source: Clin Epigenetics. 2019 Jul 1;11:96. doi: 10.1186/s13148-019-0687-0 (PMC6604333; doi:10.1186/s13148-019-0687-0)
Supplement: Supplementary file 9 — Table of primer and assay information for HIF3A.1 and HIF3A.2. (DOCX 13 kb) [file 13148_2019_687_MOESM9_ESM.docx]

| Additional file 9. Primer and assay information. | | | | | |
| --- | --- | --- | --- | --- | --- |
| Region | **Length (bp)** | **Genomic position (hg38)** | **Forward Primer** | **Reverse Primer** | **# of measurable CpG units (CpG sites)** |
| *HIF3A.1* | 338 | chr19:46,298,243-46,298,580 | 5’-AGGTTTTGGTTTTGGGTTTAATAAG-3’ | 5’-TAAAATAACAACCAACCCCAACTAA-3’ | 6 units (11 sites) |
| *HIF3A.2* | 333 | chr19:46,303,864-46,304,196 | 5’-GGGGTTTTGTTAGAATAAGGTTTT-3’ | 5’-AATCACCACCAATAAATACTACCCA-3’ | 13 units (20 sites) |
| Both forward primers had a balance tag (5’-AGGAAGAGAG-3’) added and both reverse primers had a T7 tag (5′- CAGTAATACGACTCACTATAGGGAGAAGGCT-3′) added. | | | | | |
